# Glycodelin is a potential novel follow-up biomarker for malignant pleural mesothelioma

**DOI:** 10.18632/oncotarget.12474

**Published:** 2016-10-04

**Authors:** Marc A. Schneider, Thomas Muley, Nicolas C. Kahn, Arne Warth, Michael Thomas, Felix J.F. Herth, Hendrik Dienemann, Michael Meister

**Affiliations:** ^1^ Translational Research Unit, Thoraxklinik at University Hospital Heidelberg, Roentgenstraße, Heidelberg, Germany; ^2^ Institute of Pathology, University of Heidelberg, Heidelberg, Germany; ^3^ Department of Thoracic Oncology, Thoraxklinik at University Hospital Heidelberg, Roentgenstraße, Heidelberg, Germany; ^4^ Department of Pneumology and Critical Care Medicine, Thoraxklinik at University Hospital Heidelberg, Roentgenstraße, Heidelberg, Germany; ^5^ Department of Surgery, Thoraxklinik at University Hospital Heidelberg, Roentgenstraße, Heidelberg, Germany; ^6^ Translational Lung Research Center Heidelberg, Member of the German Center for Lung Research, Heidelberg, Germany

**Keywords:** MPM, glycodelin, SMRP, biomarker, follow-up

## Abstract

Malignant pleural mesothelioma (MPM) is a rare and aggressive tumor with a short survival time arising from the mesothelial cells of the pleura. Soluble mesothelin-related peptide (SMRP), osteopontin or EFEMP1 (Fibulin-3) are well described biomarkers for malignant mesothelioma with moderate sensitivity and specificity. In this study, we characterized the expression of glycodelin, a marker for risk pregnancy, in MPM by RNA and protein analyses and investigated its potential as a MPM biomarker. We were able to detect glycodelin in the serum of MPM patients. Compared to benign lung diseases, the serum levels were significant increased. Patients with high glycodelin serum levels revealed a worse overall survival. The glycodelin serum levels correlated with the tumor response to treatment. A comparison of SMRP and glycodelin serum measurement in a large patient cohort demonstrated that the detection of both soluble factors can increase the reliable diagnostic of MPM. Glycodelin was highly expressed in MPM tumors. Analyses of a tissue micro array indicated that the immunomodulatory form glycodelin A was expressed in MPM and correlated with the survival of the patients. Altogether, glycodelin seems to be a new potential biomarker for the aggressive malignant pleural mesothelioma.

## INTRODUCTION

Malignant pleural mesothelioma (MPM) is a rare, but aggressive disease with poor prognosis [1, 2]. While the predicted peak for MPM incidence is reached in the most developed countries, the worldwide peak is still arising [3]. The majority of MPM patients have a very limited life expectancy because of unresectable disease or low response rates of chemotherapy [4, 5]. Actually, anti-folate/platinum doublet is the only approved standard of care, but combined treatment modalities are the preferred option to increase survival rates in MPM patients [6, 7].

Early diagnosis of MPM is essential for favorable prognosis but only few diagnostic biomarkers are currently known for MPM [8], e.g. soluble mesothelin-related peptide (SMRP), osteopontin [9, 10] and the EGF-containing fibulin-like extracellular matrix protein 1 (EFEMP1), also known as Fibulin-3 [11]. Mesothelin is a cell surface glycoprotein that is normally expressed at low levels in cells of mesothelium but overexpressed in several tumors, including pancreatic and ovarian adenocarcinoma, sarcomas and MPM [12]. Osteopontin is an extracellular cell adhesion protein that has been implicated in regulating metastatic spread of tumor cells [13]. The EGF containing fibulin like extracellular matrix protein 1 (EFEMP1) contains epidermal growth factor-like repeats [14]. Regarding prognosis, several studies with Receiver Operating Characteristic (ROC) curves exhibited that SMRP seems to be the most promising biomarker candidate [12, 15, 16]. A combination of biomarkers could increase the sensitivity and specificity in the MPM diagnosis [17–19].

Glycodelin, an endometrial protein (gene name *PAEP*), is well characterized during menstruation cycle and pregnancy [20]. It mediates the invasion of the trophoblast into the decidua [21, 22] and the protection of the trophoblast against the maternal immune system [23]. The latter is mediated by the immune suppressive form glycodelin A [24]. In recent years, data have shown an involvement of glycodelin in several tumors including ovarian cancer, breast cancer, and melanoma [25–27]. Glycodelin mRNA was overexpressed in non-small cell lung cancer (NSCLC) and considered a useful serum biomarker for monitoring the clinical follow-up of treated patients [28]. The purpose of the current study was to investigate whether glycodelin is expressed and secreted by MPM and whether it might be used as a novel biomarker for early diagnosis of MPM and monitoring of tumor response to treatment.

## RESULTS

### Glycodelin was detectable in the serum of patients with MPM

**Figure 1:**
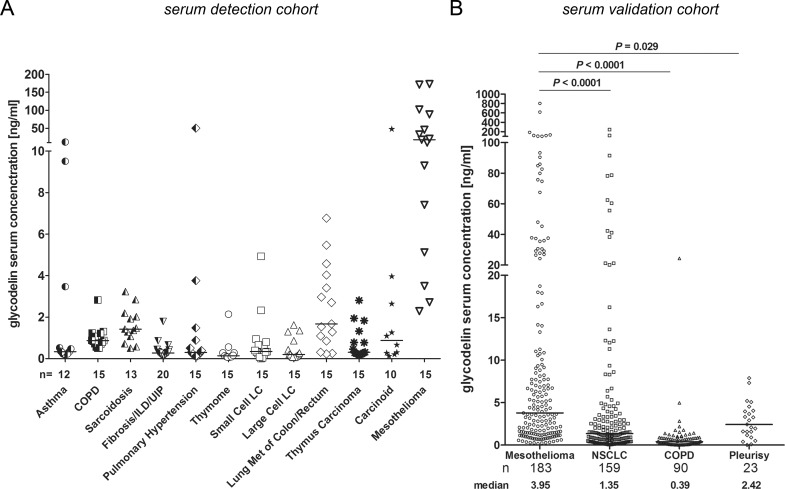
Detection of glycodelin in serum of patients with lung diseases **A.**, detection of glycodelin in serum of patients with different benign and malignant lung diseases. **B.**, comparison of glycodelin serum levels in mesothelioma, NSCLC and benign inflammatory lung diseases (please note that the NSCLC and the COPD cohorts are described elsewhere (22)). ILD = Interstitial Lung Diseases, UIP = Usual Interstitial Pneumonia, LC = Lung Cancer, Met = Metastasis, NSCLC = Non-small Cell Lung Cancer, COPD = Chronic Obstructive Pulmonary Disease.

We investigated the presence of glycodelin in serum of patients with benign and malignant thoracic diseases (Figure 1A). Most patients with benign or malignant lung diseases did not show increased glycodelin serum concentrations except patients with MPM. We confirmed the results of the detection cohort in a large validation cohort of 183 randomly selected MPM patients (Figure 1B and Table 1). The median of glycodelin in the serum of MPM patients was significantly increased compared to NSCLC patients (*P* < 0.0001), patients with COPD (*P* < 0.0001) or pleurisy (*P* = 0.029).

**Table 1:** Patients' characteristics

1. Serum validation cohort			2. Pleurisy cohort			3. TMA cohort			4. Pretherapeutical serum cohort		
Parameter	n	(%)	Parameter	n	(%)	Parameter	n	(%)	Parameter	n	(%)
*Median Age*	66 (25-88)		*Median* *Age*	69 (38-78)		*Median Age*	64 (36-87)		*Median Age*	67 (25-88)	
											
*Gender*	**183**	**100**	*Gender*	**23**	**100**	*Gender*	**213**	**100**	*Gender*	**151**	**100**
Male	163	89	Male	21	91	Male	197	92	Male	132	87
Female	20	11	Female	2	9	Female	16	8	Female	19	13
*Histology*			*Smoking status*			*Histology*			*Histology*		
E	131	72	smoker	5	22	E	166	78	E	109	72
S	22	12	nonsmoker	7	30	S	10	5	S	20	13
B	22	12	neversmoker	5	22	B	34	16	B	19	13
Other	8	4	n.d.	6	26	Other	2	1	Other	3	2
*cStage*			*asbestos contact*			*cStage*			*cStage*		
I	34	19	yes	7	30	I	59	28	I	34	23
II	42	23	no	5	22	II	90	42	II	42	28
III	55	30	n.d.	11	48	III	50	23	III	55	36
IV	23	13				IV	13	6	IV	20	13
n.d.	29	16									
*Therapy*						*Therapy*			*Therapy*		
OP	13	7				OP	7	3	OP	5	3
CT	100	55				CT	82	38	CT	88	58
RT	3	2				RT	1	0	RT	1	1
OP/CT	17	9				RT/CT	6	3	OP/CT	13	9
OP/RT	1	1				OP/CT	21	10	RT/CT	4	3
RT/CT	7	4				OP/RT	4	2	OP/RT/CT	17	11
OP/RT/CT	19	10				OP/RT/CT	50	23	no therapy	23	15
no therapy	23	13				no therapy	42	20			
5. qPCR MPM cohort			5. qPCR control cohort								
**Parameter**	**n**	**(%)**	**Parameter**	**n**	**(%)**						
*Median Age*	60 (37-76)		*Median* *Age*	72 (55-83)							
											
*Gender*	***78***	**100**	*Gender*	11	**100**						
Male	58	74	Male	4	36						
Female	20	26	Female	7	64						
											
*Histology*			*Disease*								
E	61	78	NSCLC	5	45						
S	1	1	SCLC	2	18						
B	16	21	COPD	1	9						
			other	3	27						
*pStage*											
I	0	0									
II	5	6									
III	60	77									
IV	6	7									
n.d.	*7*	9									
											
*Therapy*											
neoadj. CT/RT	64	82									
adj. CT/RT	8	10									
no CT/RT	6	8									

The pretherapeutical serum cohort is a part of the serum validation cohort. TMA = Tissue micro array, OP = surgery, CT = chemotherapy, RT = radiotherapy, E = epitheloid, S = sarcomatoid, B = biphasic, n.d. = no data

### A combination of glycodelin and SMRP can improve the prognostic values

**Figure 2:**
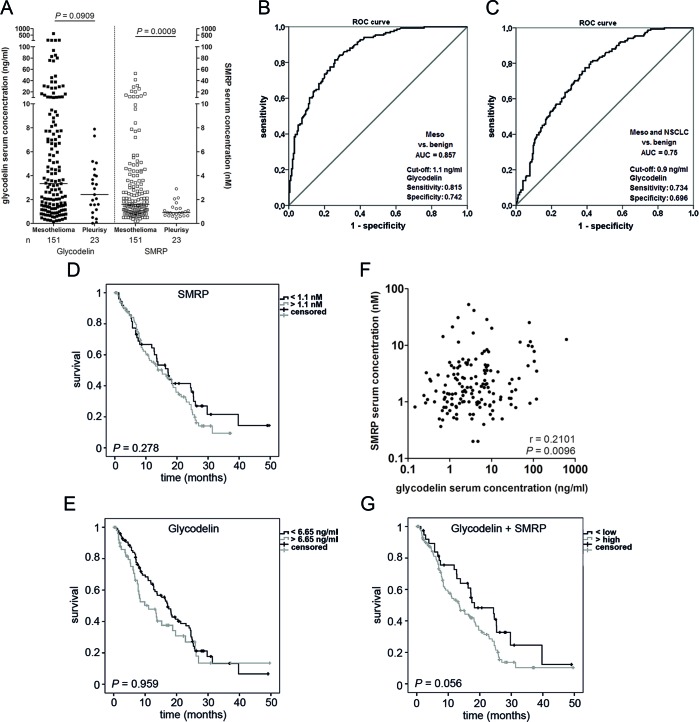
Glycodelin *vs* **SMRP** **A.**, Glycodelin and SMRP serum concentrations in patients with mesothelioma and pleurisy. **B.** and **C.**, Receiver operating characteristic (ROC) analyses with glycodelin in MPM and NSCLC. The benign cohort included all benign cases of figure 1A and B. **D.**, **E.** and **G.**, survival analyses of the pretherapeutic cohort depending on glycodelin or SMRP serum concentration and a combination of both. **F.**, correlation analyses of glycodelin and SMRP of the pretherapeutic cohort. SMRP = Soluble Mesothelin-Related Peptides

We compared the glycodelin serum concentrations with SMRP, a MPM biomarker, in a large cohort of previously untreated patients (*n* = 151, Figure 2A and Table 1) and in patients with pleurisy (*n* = 23). Approximately one third of both markers revealed low serum concentrations (< 1.5 nM or ng/ml, respectively). Nevertheless, glycodelin and the SMRP serum concentrations were increased compared to serum of patients with pleurisy (Figure 2A, *P* = 0.0909 and *P* = 0.009). Multivariate Cox-Regression analyses displayed that the age and the pathological stage were significant factors for the overall survival (Table 2). Glycodelin was only a weakly significant factor (*P* = 0.074) with a slightly increased hazard ratio (HR = 1.000-1.007) while SMRP showed no significance for the survival of the patients (*P* = 0.340, HR 0.986-1.043). In an univariate analysis regarding the MPM histologies, glycodelin was a nearly significant prognostic marker only for the epithelioid form of the MPM (*P* = 0.058). ROC analyses of the glycodelin serum concentrations in MPM vs. the benign diseases investigated in Figure 1A and 1B resulted in an area under the curve (AUC) of 0.857 (95% CI = 0.819-0.896) (Figure 2B). With additional consideration of the NSCLC samples, the AUC decreased to a value of 0.75 (95% CI = 0.704-0.797) (Figure 2C). Neither glycodelin nor SMRP levels alone were significant factors for the overall survival (OS) (Figure 2D and 2E). There was only a weak correlation between glycodelin and SMRP serum concentrations (r = 0.21, Figure 2F). A combination of both factors strongly increased the prognostic value (Figure 2G). Patients with a high serum concentration of one or both factors indicated a borderline significance for OS (*P* = 0.056) compared to patients with low serum concentrations. In further analyses this trend could be attributed mainly to patients with the epithelial MPM type (Figure S1, *P* = 0.037).

**Table 2:** Statistical analyses

Multivariate Cox-Regression analysis of overall survival			Glycodelin univariate Cox-Regression analysis of overall survival		
Variable	Hazard Ratio (95% CI)	*P*	Histology	Hazard Ratio (95% CI)	*P*
Sex	0.848 (0.453-1.588)	0.606	Epitheloid	1.003 (1.000-1.007)	0.058
Age	1.028 (1.004-1.053)	**0.022**	Sarcomatoid	1.014 (0.955-1-076)	0.645
Histology	1.098 (0.988-1.220)	0.082	Biphasic	0.942 (0.796-1.116)	0.491
pstage	1.255 (1.003-1.570)	**0.047**	n.s.	1.064 (0.849-1.335)	0.590
SMRP	1.014 (0.986-1.043)	0.340			
Glycodelin	1.003 (1.000-1.007)	0.074			

### The glycodelin serum concentrations correlate with the patients' follow-up

**Figure 3:**
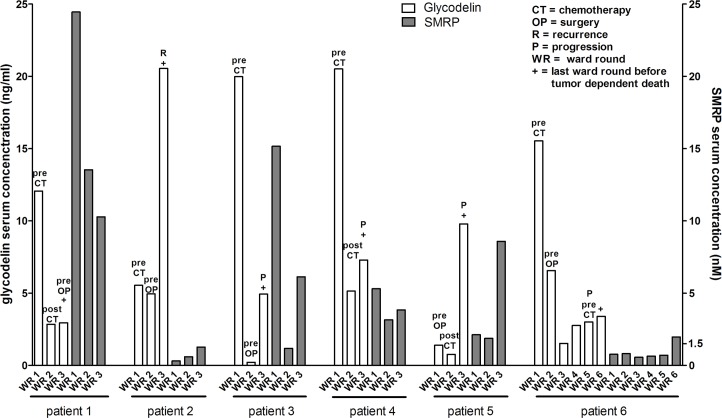
Glycodelin in MPM follow up serum samples Comparison of glycodelin and SMRP serum concentrations before treatment and during the clinical follow up of six patients with an epithelial MPM. SMRP = Soluble Mesothelin-Related Peptides.

To investigate whether glycodelin might be a valuable marker for monitoring the disease follow-up of MPM, we measured glycodelin serum concentrations in initial and follow-up samples. Examples are shown in Figure 3. The time points of serum collection are given in Table S1. In general, the pretherapeutic glycodelin level was higher than after first therapy and frequently increased during the follow-up until tumor dependent death of the patients.

We tested the same samples for the SMRP concentrations in parallel. For the patients 3, 4 and 5, we received similar results concerning the correlation of the serum concentrations and the clinical follow-up of the patients for SMRP and glycodelin. For the patients 2 and 6, the serum levels of SMRP strongly differed from the clinical disease follow-up and the glycodelin serum concentrations. Furthermore, the SMRP levels were below the manufacturers provided cut off of 1.5 nM.

### The glycodelin mRNA is overexpressed in malignant pleural cells

**Figure 4:**
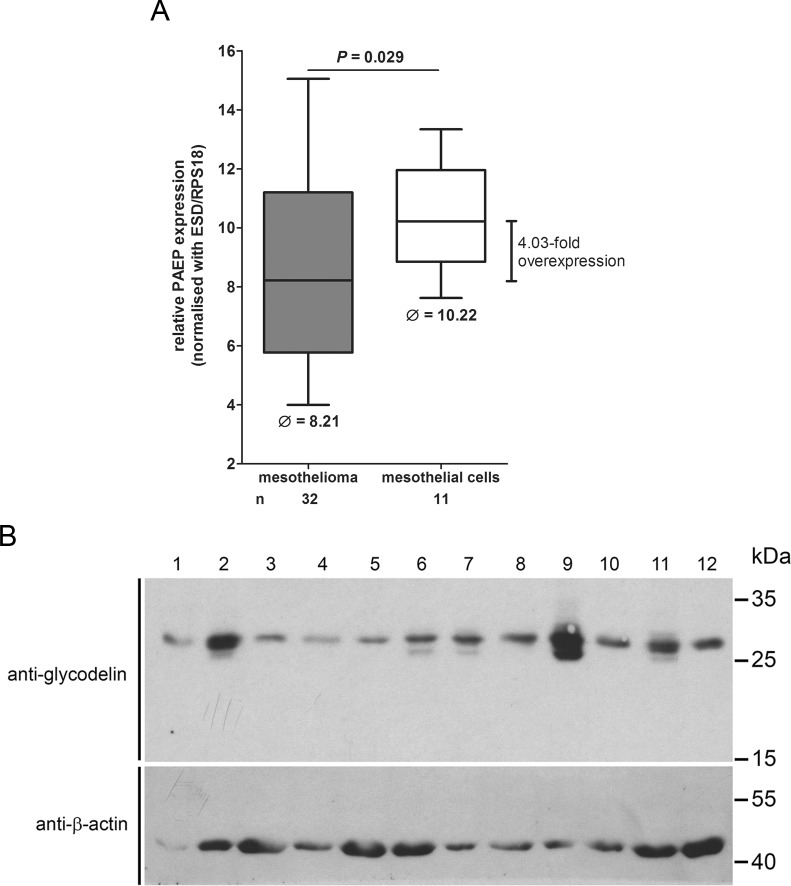
Glycodelin gene (*PAEP*) and protein expression in MPM **A.**, relative *PAEP* expression in patients with MPM compared to non-malignant mesothelial cells. Please note that a higher Ct value indicates a lower gene expression. **C.**, glycodelin protein expression in 12 primary homogenized MPM tissues. β-actin was used as a loading control.

The serum data indicated that glycodelin is expressed by the malignant cells. To validate this thesis, we analyzed the glycodelin gene expression in MPM (*n* = 32) and benign mesothelial cells (*n* = 11) derived from pleural effusions (Figure 4A and Table 1). The relative expression level in MPM was significant higher (*P* = 0.029) than in nonmalignant cells. The gene *PAEP* was 4.03-fold upregulated in the malignant cells. The relative expression level of *PAEP* in MPM was dependent on the tumor content (Figure S4). Western Blot analyses of 12 randomly selected homogenized MPM displayed an expression of glycodelin in all patients (Figure 4B).

### Glycodelin as well as glycodelin A are strongly expressed in MPM tissue

**Figure 5:**
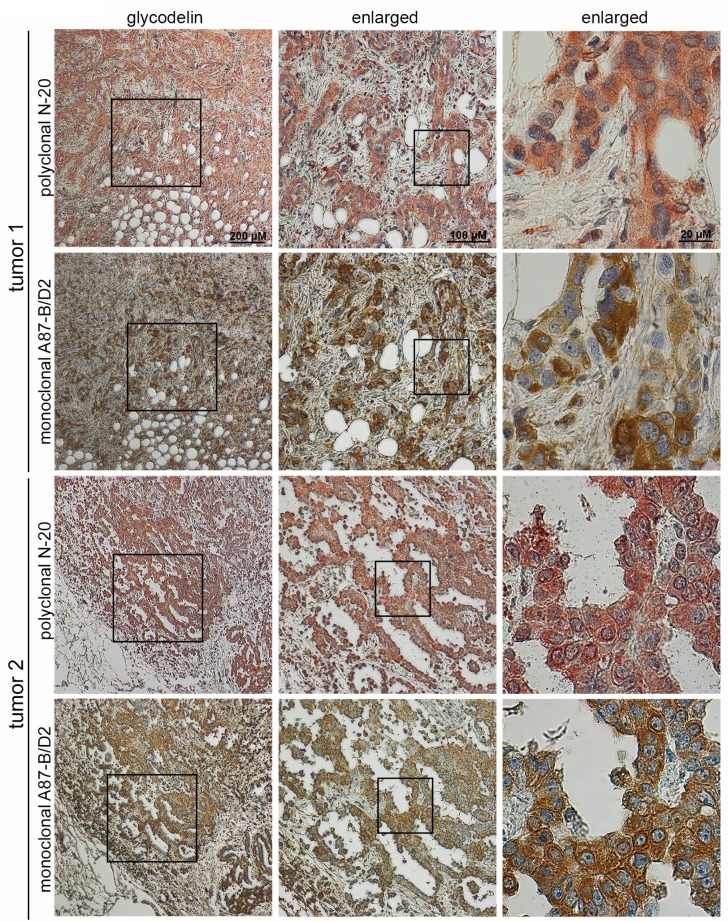
Glycodelin staining in MPM Staining of glycodelin in two representative tumours with a polyclonal glycodelin and a monoclonal glycodelin A antibody.

We performed immunohistochemistry and stained formalin-fixed and paraffin-embedded (FFPE) MPM tissue to investigate the expression of glycodelin and glycodelin A. Two representative samples are shown in Figure 5. Both tumors were resected without neoadjuvant treatment and strongly expressed glycodelin (N-20-antibody) as well as its immunosuppressive form glycodelin A (A87-B/D2-antibody). Glycodelin was expressed in the cytoplasm of the tumor cells. For tumor 1, the staining pattern of the two antibodies was heterogeneous. While the polyclonal antibody stained nearly all tumor cells, the immunosuppressive form glycodelin A was only partly and inhomogeneously expressed within the tumor nests (see enlarged areas). Antibody specificity was tested and validated in controls (Figure S2) and elsewhere [29].

### A high glycodelin A expression in MPM tissue indicates a benefit for the survival of the patients

**Figure 6:**
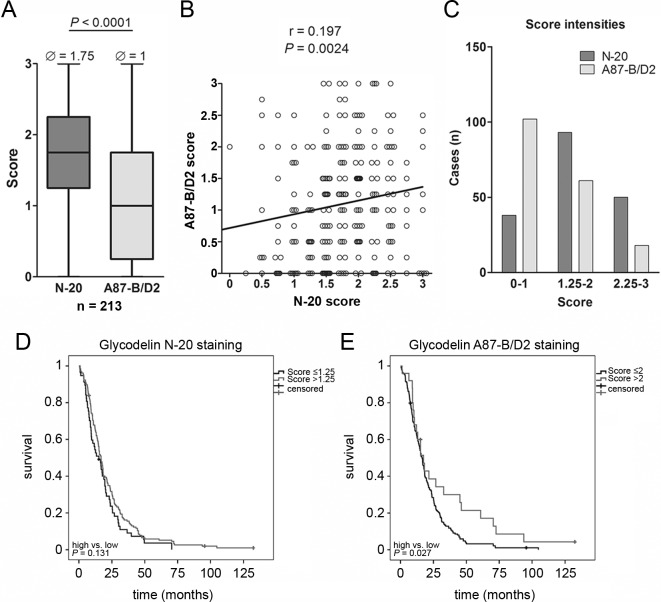
Analyses of a MPM tissue microarray (TMA) **A.**, results of TMA scoring with either the polyclonal N-20 or the monoclonal A87-B/D2 glycodelin antibody stained mesothelioma patients (n = 214). **B.**, correlation between N-20 and A87-B/D2 stainings. **C.**, distribution of scoring. **D.** and **E.**, survival analyses depending on glycodelin staining intensities and antibodies.

To further investigate the prognostic value of glycodelin, we stained a MPM tissue microarray (*n* = 213, see also Table 1) for glycodelin as well as for glycodelin A. A scoring was performed to analyze the expression levels of glycodelin (Figure 6 and S3A). In general, the total glycodelin antibody displayed a significant stronger staining compared to the glycodelin A specific antibody (Figure 6A, *P* < 0.0001). The correlation between the staining intensities was low (Figure 6B, r = 0.197). While the majority of stainings with the N-20 antibody reached a scoring between 1.25 and 3, most of the samples stained with the glycodelin A specific antibody exhibited a scoring between 0 and 2 (Figure 6C). Survival analyzes revealed for both antibodies that a higher score correlated with a better OS of the patients. While the total glycodelin failed to be a significant prognostic marker (Figure 6D, *P* = 0.131), a strong glycodelin A staining resulted in a significant better OS (Figure 6E, *P* = 0.027). Further analyses indicated that only males (*P* = 0.032 for glycodelin and *P* = 0.035 for glycodelin A) but not females (*P* = 0.185 and *P* = 0.488) exhibited a better survival (Figure S3 B-E).

## DISCUSSION

We demonstrated for the first time the expression of glycodelin mRNA and protein in MPM and provided first data concerning its potential as a prognostic and clinical marker for this cancer entity. Since MPM is an aggressive malignancy with poor prognosis, rapid progression and limited therapeutic options [1], novel biomarkers which correlate with the tumor response to treatment are highly warranted.

Glycodelin is expressed during menstruation cycle and pregnancy but also in the hormone-related cancers such as breast and ovarian cancer [30]. In a previous study, we showed that glycodelin was expressed in NSCLC tumors and can be used as a biomarker for monitoring of the tumor burden during the therapy [28].

In a serum test cohort of patients with benign and malignant lung diseases, we measured increased glycodelin concentrations especially in patients with MPM. A large validation cohort exhibited that the glycodelin levels were significant higher than in benign diseases such as COPD and pleurisy but also compared to patients with NSCLC. ROC analyses indicated that glycodelin reached AUC-values that were comparable or better than the markers SMRP, EFEMP1 (fibulin-3) or osteopontin [15, 19, 31]. Glycodelin might therefore be used as a supportive biomarker for MPM in the differential diagnostic settings. However, since glycodelin was also expressed by NSCLC, the ROC analyses combining MPM and NSCLC vs. benign diseases showed lower specificity and sensitivity. Surprisingly, our data showed that the glycodelin serum concentration and the SMRP serum concentration did not correlate. Therefore, a combination of glycodelin with another MPM marker, i.e. SMRP, could increase the prognostic value since SMRP alone was also not prognostic in our cohort.

The majority of MPM results from a continuous inflammation that is often caused by asbestos fibers on the surface of the pleura with an attributable risk of 87.3 % for men and 64.8% for women [32]. To differentiate between benign and malignant mesothelial proliferations can still be a diagnostic challenge [33]. Our data show that the glycodelin serum concentrations were elevated, but not significant in patients with MPM compared to pleurisy. There were several patients within the pleurisy cohort that revealed increased glycodelin levels. Unfortunately, the follow-up of a part of the patients was not performed in our hospital to clarify the disease progression. Nevertheless, in our pleurisy cohort, the patient with the highest glycodelin serum concentration, a never smoker with chronical pleurisy after asbestos exposition, later developed a MPM, pointing towards the potential diagnostic use of glycodelin for early detection of MPM.

Since MPM is an aggressive tumor, some patients undergo an initial chemotherapy to reduce the tumor burden, followed by a surgery or/and radiotherapy. Therefore, a closely follow-up is indispensable to detect a recurrence or growth of the tumor at an early stage. We measured the glycodelin serum concentrations during the clinical follow-up of the MPM patients and observed a strong correlation between the serum levels and the tumor response to treatment. A comparison with SMRP serum concentrations indicated that glycodelin might be a more specific biomarker for the follow-up measurements. Further studies with more frequent glycodelin serum monitoring shall clarify whether the glycodelin concentration might earlier indicate a growth of tumor burden or metastatic disease compared to other clinical diagnostics procedures.

Although glycodelin was expressed on protein level in all 12 homogenized tumors and we noted a higher gene expression in MPM compared to healthy tissue, there were also several patients with a low glycodelin concentration in the validation cohort. One reason might be that the glycodelin serum concentration depends on several factors such as tumor size, tumor vascularization or metastatic disease. This fits with the observation we made in the NSCLC cohort, where *PAEP* was overexpressed in more than 80% of all tumors but was much less detectable in sera of pretherapeutical NSCLC patients [28].

In MPM, we detected both, total glycodelin as well as the immunosuppressive form glycodelin A. The correlation of both staining patterns was low and the generally reduced expression of the immunosuppressive form glycodelin A indicated that both antibodies differentiated probably well between the glycodelin forms. Survival analyses of staining intensities showed a similar trend. A significant effect for OS was observed only for the expression of glycodelin A. In contrast to the situation in NSCLC, glycodelin and especially glycodelin A expression levels in the tumor seemed to have a positive effect on OS of the MPM patients. The reason for this observation is unclear. Since MPM are mainly a result of a chronic inflammation, the expression of glycodelin A might reduce the inflammation between the pleural layers. The slightly elevated serum concentrations in patients with pleurisy might support this theory. Many studies showed that glycodelin A suppresses the activity of immune cells [23, 34, 35]. These findings do not contrast inevitably with the prognostic results from serum measurements. There, a high serum concentration obviously indicated a high tumor load. In future studies we will isolate glycodelin from NSCLC and MPM to co-cultivate immune cells with the tumor-derived glycodelin. These experiments shall clarify its role within the tumor immune system interface.

In summary, our data strongly suggest that glycodelin might be a feasible serum marker for the diagnosis of MPM and the monitoring of tumor response to treatment during the follow-up of MPM patients.

## MATERIALS AND METHODS

### Biomaterial collection, characterization and preparation

Tissue, blood and pleural effusion samples were provided by the Lung Biobank Heidelberg, a member of the accredited Tissue Bank of the National Center for Tumor Diseases (NCT) Heidelberg, the BioMaterialBank Heidelberg, and the Biobank platform of the German Center for Lung Research (DZL). All patients provided written informed consent for the use of their biomaterials for research purpose. The studies were approved by the local ethics committee of the University of Heidelberg. For the tissue micro array (TMA) MPM samples of 213 patients who underwent surgery between 2002 and 2009 at the Thoraxklinik at University Hospital Heidelberg, Heidelberg, Germany were collected (Table 1). Before TMA construction, a hematoxylin and eosin (H&E)-stained slide of each block was analyzed to select tumor-containing regions. A TMA machine (AlphaMetrix Biotech, Roedermark, Germany) was used to extract tandem 1.0-mm cylindrical core sample from each tissue donor block. All diagnoses were made according to the 2004 World Health Organization classification [36] for MPM by at least two experienced pathologists. Tumor histology was classified according to the 7^th^ edition of the Union internationale contre le cancer (UICC) tumor, node, and metastasis [37]. Tissues were snap-frozen within 30 minutes after resection and stored at -80°C until the time of analysis. For nucleic acid isolation 10 - 15 tumor cryosections (10 - 15 mm each) were prepared for each patient. The first and the last sections in each series were stained with H&E and reviewed by an experienced lung pathologist to determine the proportions of viable tumor cells, stromal cells, normal lung cell cells, infiltrating lymphocytes and necrotic areas [38]. Only samples with a viable tumor content of ≥ 50% were used for subsequent analyses. As a control, mesothelial cells from pleural effusions were collected (*n* = 11). Only samples with a content of ≥ 50% mesothelial cells and without tumor cells were included.

### Detection of glycodelin in human sera

Sera were collected prior to any disease-specific treatment and stored at −80°C within 2 h after venipuncture. All patients provided written informed consent for the use of the serum for research purpose. The Non-small cell lung cancer (NSCLC) and chronic obstructive pulmonary disease (COPD) cohorts were described elsewhere [28]. The MPM cohort included 214 randomly selected sera of MPM patients (see Table 1). As a reference pleural patient group, patients with a pleurisy resulting from a benign disease were included (see Table 1). The benign and malignant test cohorts of the serum detection cohort included consecutive collected sera of patients with the indicated diseases. The glycodelin levels of the sera were measured in two replicates using an enzyme-linked immunosorbent assay kit (ELISA BS-20-30, Bioserv Diagnostics, Rostock, Germany). In tumor follow-up samples, glycodelin was measured in sera collected during the patient's routine checkup and/or before repeated clinical intervention. The measurement of soluble mesothelin-related peptides (SMRP) was performed using the MESOMARK^®^ ELISA (Fujirebio Diagnostics, Malvern, PA, US). The readouts and standard curves were performed with ELISA Reader (Tecan Group Ltd., Crailsheim, Germany). The results of the ELISA were visualized with GraphPad Prism 5.

### Detection of glycodelin in human tissue

For the detection of glycodelin in tissues of MPM patients, cryosections (10 - 15 mm thick) of snap frozen MPM tissues were prepared. For each 100 mg of tissue, 300 μl PBS with protease inhibitors (10 ng/ml Aprotinin, 100 μM Leupeptin, 1 μM Pepstatin, 1 mM PMSF, all Carl Roth, Karlsruhe, Germany) was added. Cryosections were homogenized with the TissueLyser mixer-mill disruptor (1 min, 25 Hz, Qiagen, Hilden, Germany) followed by a centrifugation step for 10 min with 13000 x g at 4°C. The supernatants of the samples were used for glycodelin detection by Western blot. Glycodelin was detected with the polyclonal N-20 antibody (sc-12289, Santa Cruz Biotechnology, Heidelberg, Germany). Anti-beta-actin (#A5441 Sigma-Aldrich) was used as a loading control.

### Total RNA isolation, cDNA synthesis, and quantitative Real-Time PCR (qPCR)

RNA isolation, cDNA synthesis and quantitative Real-Time PCR (qPCR) were performed as described elsewhere [28].

### Immunohistochemistry

Glycodelin immunohistochemistry was performed as described elsewhere [28]. Pictures were taken with an Olympus Color View II digital camera and Olympus Cell-F software (Olympus).

### Statistical analyses

Data of serum and TMA analyses were statistically analyzed under REMARK criteria [39] with SPSS 22.0 for Windows (IBM, Ehningen, Germany). The evaluation of discriminatory values for glycodelin expression in tumor and for glycodelin serum concentrations that best differentiated between groups of patients with good and poor survival prognosis was performed with the critlevel procedure using ADAM statistical software package [40] (German Cancer Research Center, DKFZ, Heidelberg, Germany). Binary variables were built using these cut-offs. The endpoint of the study was overall survival (OS). Survival was calculated from the date of surgery until the last date of contact or death. Multivariate survival analysis was performed using the Cox proportional hazards model. Univariate analysis of survival data was performed according to Kaplan and Meier [41] and using Cox proportional hazard models. The log-rank test was used to test the significance between the groups. A p-value of less than 0.05 was considered significant. ELISA data were statistically analyzed with GraphPad Prism 5. The non-parametric Mann-Whitney U test [42] as well as the Kruskal-Wallis test [43] were used to investigate significant differences between the patient groups.

## SUPPLEMENTARY MATERIALS FIGURES AND TABLE


