# Supplementary material for: Glycodelin is a potential novel follow-up biomarker for malignant pleural mesothelioma
Source: Oncotarget. 2016 Oct 4;7(44):71285–97. doi: 10.18632/oncotarget.12474 (PMC5342078; doi:10.18632/oncotarget.12474)
Supplement: Supplementary file 1 [file oncotarget-07-71285-s001.pdf]

Glycodelin is a potential novel follow-up biomarker for malignant pleural mesothelioma

Supplementary Material

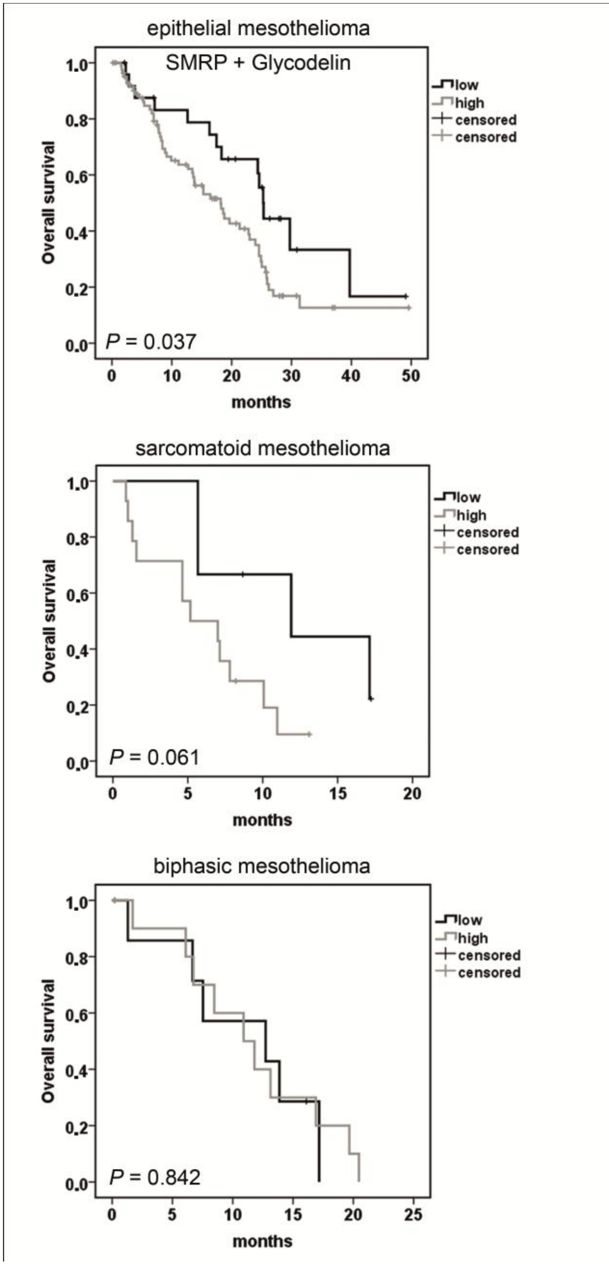

Supplementary Figure 1: Survival analyses of the glycodelin pretherapeutical cohort

Survival analyses of the pretherapeutic cohort with glycodelin and SMRP detection in combination. "Low" means concentrations below the indicated concentrations of Fig 2 D and E, "high" means one or both protein serum concentrations above the indicated concentrations. SMRP = Soluble Mesothelin-Related Peptides

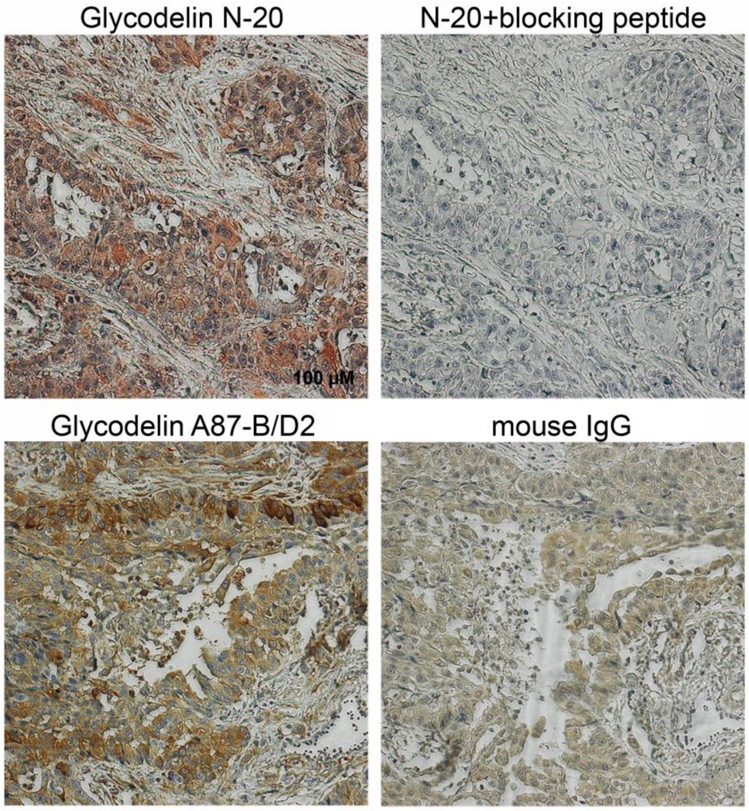

Supplementary Figure 2: Immunohistochemistry antibody controls

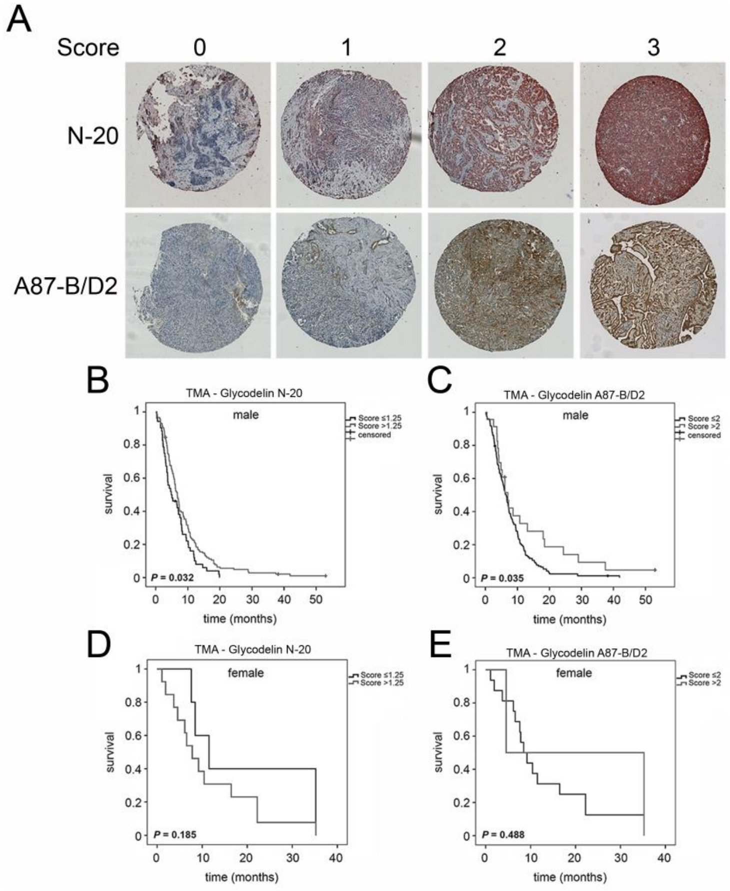

Supplementary Figure 3: TMA scoring. A., scoring examples for the two glycodelin antibodies N-20 and A87-B/D2. **B.- E.**, survival analyses of patients depending on glycodelin staining intensities and antibodies. TMA = Tissue Micro Array

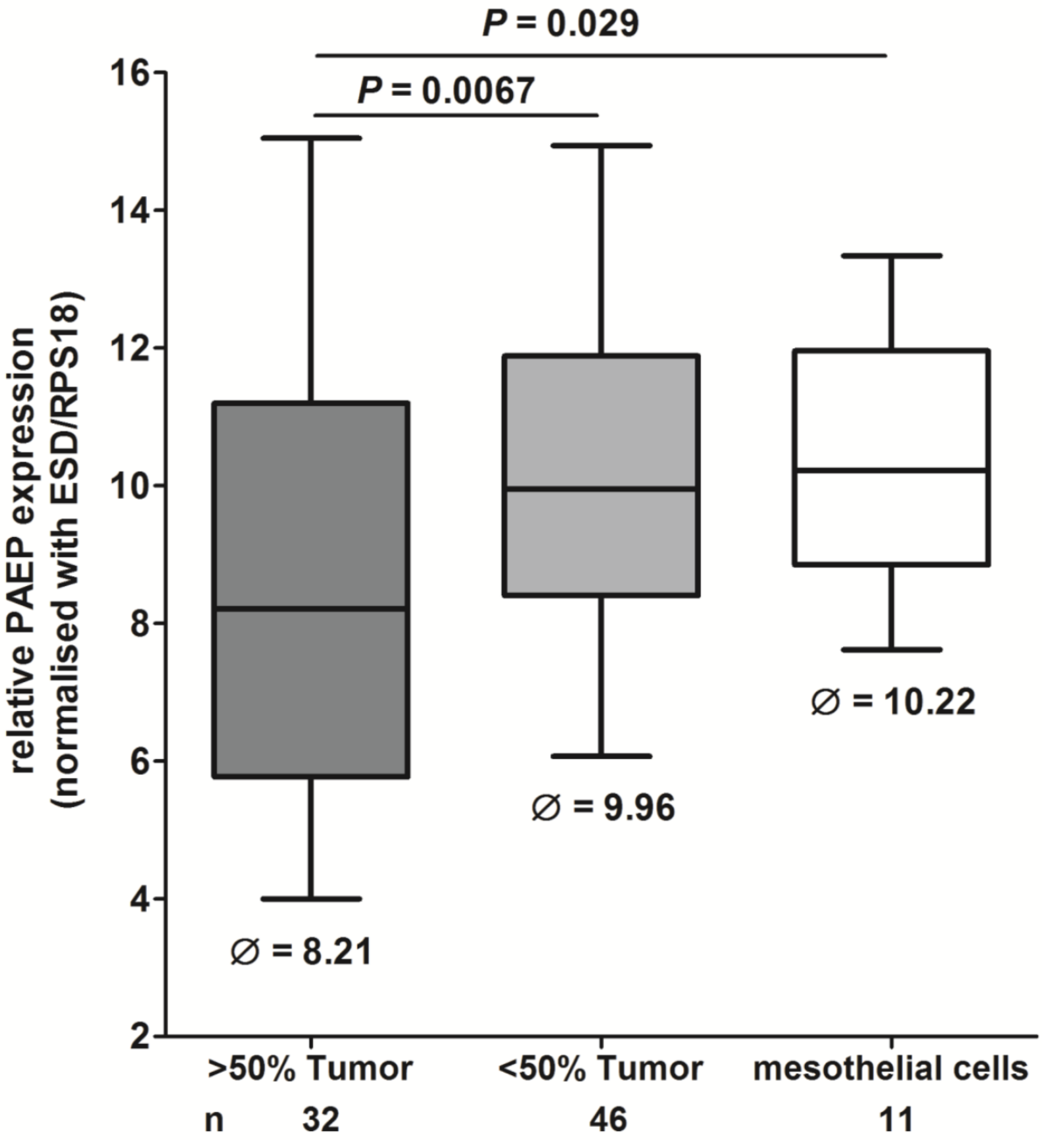

Supplementary Figure 4: Additional qPCR results. Relative *PAEP* expression in samples with higher tumor content, lower tumor content and non-malignant mesothelial cells. Please note that a higher Ct value indicates a lower gene expression.

Table S1. Patient blood collection intervals

patient	ward round	intervall (months)
1	1	-
	2	4
	3	1
2	1	-
	2	4
	3	14
3	1	-
	2	4
	3	8
4	1	-
	2	15
	3	3
5	1	-
	2	11
	3	54
6	1	-
	2	3
	3	5
	4	3
	5	3
	6	10
